# Supplementary material for: The ∆Pv-aCO2/∆Ca-vO2 ratio as a predictor of mortality in patients with severe acute respiratory distress syndrome related to COVID-19
Source: PLoS One. 2023 Sep 6;18(9):e0290272. doi: 10.1371/journal.pone.0290272 (PMC10482256; doi:10.1371/journal.pone.0290272)
Supplement: Attachment — Submitted filename: Response to Reviewers.docx [file pone.0290272.s001.docx]

January 20^th^, 2023

**Emily Chenette**

**Editor-in-Chief**

**Bora Çekmen**

**Academic Editor**

**PlosONE**

Dear Editor,

Enclosed, please find the revised version of the manuscript entitled: “**∆Pv-aCO2/∆Ca-vO2 ratio as predictor of mortality in patients with acute respiratory distress syndrome related to COVID-19**” with ID: **PONE-D-22-29034**, which we are submitting for consideration for publication in PLOS ONE.

We attended to all the suggestions of the reviewers and performed additional modifications to complete this work.

Journal Requirements:

When submitting your revision, we need you to address these additional requirements.

1. Please ensure that your manuscript meets PLOS ONE's style requirements, including those for file naming. The PLOS ONE style templates can be found at

https://journals.plos.org/plosone/s/file?id=wjVg/PLOSOne_formatting_sample_main_body.pdf and

<https://journals.plos.org/plosone/s/file?id=ba62/PLOSOne_formatting_sample_title_authors_affiliations.pdf>.

***Reply:*** *Thank you very much for your comments. In this new submission, we ensured that our manuscript met PLOS ONE´s style requirements. The Postal Codes were removed from the affiliations, and the list of corresponding authors was modified, including the initials in parentheses after the email address.*

2. Please include your tables as part of your main manuscript and remove the individual files. Please note that supplementary tables (should remain/ be uploaded) as separate "supporting information" files"

***Reply:*** *Tables 1 and 2 were included as part of the main manuscript (Pages 18-20), and the individual files were removed.*

3. Please review your reference list to ensure that it is complete and correct. If you have cited papers that have been retracted, please include the rationale for doing so in the manuscript text, or remove these references and replace them with relevant current references. Any changes to the reference list should be mentioned in the rebuttal letter that accompanies your revised manuscript. If you need to cite a retracted article, indicate the article’s retracted status in the References list and also include a citation and full reference for the retraction notice.

***Reply:*** *References have been checked, and we have ensured they are complete and correct. In addition, we changed some of the references to more current ones, as shown below:*

*The reference: “12. Mallat J, Lemyze M, Tronchon L, Vallet B, Thevenin D. Use of venous-to-arterial carbon dioxide tension difference to guide resuscitation therapy in septic shock. World J Crit Care Med. 2016; 5(1):47-56. https://doi.org/10.5492/wjccm.v5.i1.47 PMID: 26855893”* ***was changed to*** *“12.Nassar B, Badr M, Van Grunderbeeck N, Temime J, Pepy F, Gasan Gaelle, et al. Central venous-to-arterial PCO2 difference as a marker to identify fluid responsiveness in septic shock. Sci Rep. 2021; 11(1):17256. https://doi.org/10.1038/s41598-021-96806-6 PMID: 34446823”*

*The reference: “15. Dubin A, Pozo MO, Hurtado J. Venoarterial PCO2-to-arteriovenous oxygen content difference ratio is a poor surrogate for anaerobic metabolism in hemodilution: an experimental study. Rev Bras Ter Intensiva. 2020; 32(1):115-122. https://doi.org/10.5935/0103-507x.20200017 PMID: 32401981”* ***was changed to*** *“15. Dubin A, Ferrara G, Edul VSK, Martins E, Canales HS, Canullán C, et al. Venoarterial PCO2-to-arteriovenous oxygen content difference ratio is a poor surrogate for anaerobic metabolism in hemodilution: an experimental study. Ann Intensive Care. 2017; 7(1):65. https://doi.org/10.1186/s13613-017-0288-z PMID: 28608134”*

*The reference: “16. Zubieta-Calleja G, Zubieta-DeUrioste N. Pneumolysis and "Silent Hypoxemia" in COVID-19. Indian J Clin Biochem. 2020 Nov 9;36(1):1-5. doi: 10.1007/s12291-020-00935-0”* ***was changed to*** *“16. Al-Kuraishy HM, Al-Gareeb A, Al-Hamash SM, Cavalu S, El-Bouseary M, Sonbol F, et al. Changes in the Blood Viscosity in Patients With SARS-CoV-2 Infection. Front Med. 2022;17;9: 876017. https://doi.org/10.3389/fmed.2022.876017 PMID: 35783600”*

*The reference: “17. Arora S, Tantia P. Physiology of Oxygen Transport and its Determinants in Intensive Care Unit. Indian J Crit Care Med. 2019 Sep;23(Suppl 3): S172-S177. DOI: 10.5005/jp-journals-10071-23246”* ***was changed to*** *“17. Bracegirdle L, Jackson A, Beecham R, Burova M, Hunter E, Hamilton LG, et al. Dynamic blood oxygen indices in mechanically ventilated COVID-19 patients with acute hypoxic respiratory failure: A cohort study. PLoS One. 2022;17(6): e0269471. https://doi.org/10.1371/journal.pone.0269471 PMID: 35687543”*

*The reference: “20. Kattan E, Hernández G. The role of peripheral perfusion markers and lactate in septic shock resuscitation. Journal of Intensive Medicine, https://doi.org/10.1016/j.jointm.2021.11.002”* ***was changed to*** *“20. Cruces P, Retamal J, Hurtado DE, Erranz B, Iturrieta P, González C, et al. A physiological approach to understand the role of respiratory effort in the progression of lung injury in SARS-CoV-2 infection. Crit Care. 2020;24(1): 494. https://doi.org/10.1186/s13054-020-03197-7 PMID: 32778136”*

*The reference: “32. Hassanein A, Abbas I, Mohammed R. Central blood gases versus lactate level for assessment of initial resuscitation success in patients with sepsis in critical care, Egyptian Journal of Anaesthesia. 2022. 38:1, 439-445, DOI: 10.1080/11101849.2022.2108196”* ***was changed to*** *“32. McDonald CI, Brodie D, Schmidt M, Hay K, Shekar K. Elevated Venous to Arterial Carbon Dioxide Gap and Anion Gap Are Associated with Poor Outcome in Cardiogenic Shock Requiring Extracorporeal Membrane Oxygenation Support. ASAIO J. 2021;67(3): 263-269. https://doi.org/10.1097/MAT.0000000000001215 PMID: 33627599”*

**Reviewers' comments: Reviewer's Responses to Questions**

***Reply:*** *Thank you very much for your comments.*

**Comments to the Author**

1. Is the manuscript technically sound, and do the data support the conclusions?

The manuscript must describe a technically sound piece of scientific research with data that supports the conclusions. Experiments must have been conducted rigorously, with appropriate controls, replication, and sample sizes. The conclusions must be drawn appropriately based on the data presented.

Reviewer #1: Partly

Reviewer #2: Yes

2. Has the statistical analysis been performed appropriately and rigorously?

Reviewer #1: Yes

Reviewer #2: Yes

3. Have the authors made all data underlying the findings in their manuscript fully available?

The [PLOS Data policy](http://www.plosone.org/static/policies.action#sharing) requires authors to make all data underlying the findings described in their manuscript fully available without restriction, with rare exception (please refer to the Data Availability Statement in the manuscript PDF file). The data should be provided as part of the manuscript or its supporting information, or deposited to a public repository. For example, in addition to summary statistics, the data points behind means, medians and variance measures should be available. If there are restrictions on publicly sharing data—e.g. participant privacy or use of data from a third party—those must be specified.

Reviewer #1: Yes

Reviewer #2: Yes

4. Is the manuscript presented in an intelligible fashion and written in standard English?

PLOS ONE does not copyedit accepted manuscripts, so the language in submitted articles must be clear, correct, and unambiguous. Any typographical or grammatical errors should be corrected at revision, so please note any specific errors here.

Reviewer #1: Yes

Reviewer #2: Yes

**5. Review Comments to the Author**

Please use the space provided to explain your answers to the questions above. You may also include additional comments for the author, including concerns about dual publication, research ethics, or publication ethics. (Please upload your review as an attachment if it exceeds 20,000 characters)

**Reviewer #1:** Thank you for the opportunity to review this manuscript which quantifies the effect of the ratio ΔPv-aCO2 / ΔCa-vO2 on the mortality of patients with ARDS secondary to SARS-CoV-2.

Major comment:

The methodology is sound. My main issue is the concluding statement : “Patients with ARDS secondary to SARS-CoV-2 have increased ΔPv-aCO2/ΔCa-vO2 ratio …” This phrasing implies a comparison of this ratio between patients w/ ARDS secondary to SARS-CoV-2 and patients without ARDS secondary to SARS-CoV-2. That is not the case, because this study presents data only from the first group. The conclusion should be modified to simply state something to the effect of: “In patients with ARDS secondary to SARS-CoV-2, non-survivors have increased ΔPv-aCO2/ΔCa-vO2 ratio compared to the survivors …” This modified phrasing more accurately circles back to the title and stated objective of the study, and it is supported by the data and methodology presented here.

***Reply:*** *Thank you very much for your comments. We modified the concluding statement according to the reviewer's comment to refer more precisely to the title and stated objective of our study, which is supported by the data and methodology presented here.*

*The concluding statement: “Patients with ARDS secondary to SARS-CoV-2 have increased ∆Pv-aCO2/∆Ca-vO2 ratio due to hypoxia secondary to severe hypoxemia…”* ***was changed to*** *“In patients with ARDS secondary to SARS-CoV-2, non-survivors have an increased ∆Pv-aCO2/∆Ca-vO2 ratio due to hypoxia secondary to severe hypoxemia…”*

Minor comments:

**1)** The 3rd paragraph of the Results section has this statement -- “Fig 3 and Fig 4A shows [sic] the Kaplan-Meier …” But Fig 3A is the ROC curve, while 4A is the Kaplan-Meier curve for ΔPv-aCO2/ΔCa-vO2.

***Reply:*** *We agree with the reviewer that Figure 3 corresponds to the ROC curve, therefore in this new submission, this error was corrected as follows:*

*“The AUC for ∆Pv-aCO2/∆Ca-vO2 ratio was 0.691(95% CI 0.598-0.774, p= 0.0001), with a best cut-off point of >2.14 mmHg/mL (sensitivity 49.18%, specificity 85.19%, positive likelihood ratio (LR+) 3.32, and negative likelihood ratio (LR-) 0.6) (Fig 3A). The best cut-off point obtained by Youden's index for SAPS II was >74 points (AUC= 0.696 (95% CI 0.603-0.778, p= 0.0001), sensitivity 65.57%, specificity 64.81%, LR+ 1.86, and LR- 0.53) (Fig 3B). Fig 4A shows the Kaplan-Meier curve of the ∆Pv-aCO2/∆Ca-vO2 ratio for 30-day survival, showing a statistically significant difference between survivors and non-survivors with a cut-off point of >2.14 mmHg/mL.”*

**2)** I noticed a couple of grammatical errors “shows” (instead of “show”) – see [sic] above; and “shown” instead of “shows” (see end of 2nd paragraph of the Results section).

***Reply:*** *The words “shows” and “shown” were corrected according to the reviewer's comment.*

*“Table 2 show the Cox regression analysis for mortality in patients with ARDS secondary to SARS-CoV-2. In the univariate analysis, the variables BMI, smoking, Diabetes, SAPS II, vasopressor, pH, base, ∆Pv-aCO2, and ∆Pv-aCO2/∆Ca-vO2 ratio had statistical significance. In the multivariable analysis, BMI (HR 1.04, 95% CI 1.01-1.08, p= 0.007), SAPS II (HR 1.04, 95% CI 1.01-1.08, p= 0.005), and ∆Pv-aCO2/∆Ca-vO2 ratio (HR 1.17, 95% CI 1.06-1.29, p= 0.001) maintained statistical significance. Fig 2 shows the final Cox regression model for mortality in patients with COVID-19.”*

**3)** You use the two notations “Pv-aCO2” and “ΔPv-aCO2” interchangeably. If you mean for them to be interchangeable, then it would be better to just pick one and stay with it throughout the manuscript (to minimize confusion).

***Reply:*** *In this new submission, the notation “Pv-aCO2” was changed to “ΔPv-aCO2,” and this word was maintained throughout the manuscript to minimize confusion, as suggested by the reviewer.*

*“ΔPv-aCO2/ΔCa−vO2 ratio= ΔPv-aCO2/ΔCa−vO2” (Definitions, Section of Material and Methods).*

*“The correlation between ΔPv-aCO2/ΔCa-vO2 and SAPS II was calculated using the Spearman correlation test. All analyses were performed using R Studio v4.03 statistical (R Foundation, Vienna, Austria) and SPSS v.25 software (IBM, New York, USA)” (Section of Statistical analysis).*

*In addition, an error in Figure 2 was detected (“Pa-vCO2” instead of “ΔPv-aCO2”). Therefore the figure was modified and uploaded in this submission of the manuscript.*

**4)** In the Introduction section, shouldn't there be a period between “... most commonly used cut-off point [8]” and “ScvO2 surrogates …” ?

***Reply:*** *We added a period between “... most commonly used cut-off point [8]” and “ScvO2 surrogates …” as suggested by the reviewer.*

*“… most commonly used cut-off point [8]. ScvO_2_ surrogates…”*

**5)** In the Introduction, you wrote: “... but without inappropriate blood flow, CO2 will be poorly …” – this seems like an unintended double negative, so either remove "without" or change “inappropriate” to “appropriate”.

***Reply:*** *In this new submission, the sentence in the Introduction was modified by changing “inappropriate” to “appropriate” according to the reviewer's commentary:*

*“… but without appropriate blood flow, CO2 will be poorly …”*

**6)** In the Introduction, what does the acronym “CR” stand for? It appears twice without definition.

***Reply:*** *The acronym “CR” is a typo. In this sense, the correct acronym is “RQ”. This word was corrected in this new submission.*

**7)** The second to last sentence under the “Data Collection” paragraph – “Other variables ..” is incomplete.

***Reply:*** *In this new submission, the second to last sentence under the “Data Collection” paragraph – “Other variables ..” was completed as suggested by the reviewer.*

*“Other variables such as creatinine, D-dimer, C-reactive protein, fibrinogen, glutamic oxaloacetic transaminase [GOT], glutamic pyruvic transaminase [GPT], and vasopressor were also included in this study.”*

**Reviewer #2:** It was a well-considered idea to research ARDS patients with hypoxemic hypoxia. ΔPvaCO2/ΔCa-vO2 ratio might be useful to detect the mortality and prognosis of severe Covid-19 patients. But there are some concerns about the article which I stated below.

**1.** In the Introduction section the sentences start with ‘The CR will increase by higher VCO2 or lower VO2…….’, you used CR for the abbreviation, but this abbreviation was not clearly defined before.

***Reply:*** *Thank you very much for your comments.* *The acronym “CR” is a typo, the correct acronym is “RQ”. This word was corrected in this new submission as follows:*

*‘The RQ will increase by higher VCO2 or lower VO2…….’*

**2.** The authors stated that all the patient was intubated with this research. Was the PaO2/FiO2 ratio obtained before intubation or it came from after the intubation period?

***Reply****: The arterial and central venous blood gases,* *including PaO2/FiO2 ratio, were obtained 30 minutes after intubation and processed in the GEM® PREMIER™4000 with iQM® equipment. This sentence was included in this new submission (Section of Material and methods, Study design and patients).*

**3.** Why did not the authors exclude patients with hematologic disease? Many diseases might affect the hemoglobin level. It would be better to state that in the Method section.

***Reply:*** *We agree with the reviewer's comment. Because many diseases might affect the hemoglobin level as hematologic diseases, we decided to exclude patients with this characteristic from the beginning of this study. This exclusion criterion was included in this submission as follows:*

*“(1) Patients with diseases that could affect the hemoglobin level as hematologic diseases, (2) patients with an incomplete variable registry, or (3) pregnant were excluded from this study.” (Section of Material and methods, Study design and patients).*

**4.** In table 1, smoking differed between the two groups, and it is well known that smoking can increase CO2 in blood gas analysis. Due to this issue, it would be better to add a limitation to this article. Also, it was shown that all the patients were Level 3 ARDS patients (With Berlin Criteria). So, it would be better to state that this research was held with severe ARDS patients in both title and method section.

***Reply:*** *Although the variable “smoking” between the two groups had a statistically significant difference, in the multivariable COX regression analysis, this variable was not found to be an independent risk factor for mortality of patients with acute respiratory distress syndrome related to COVID-19. However, we agree with the reviewer that smoking can increase CO_2_ in blood gas analysis. Due to this issue, we decided to add a limitation to this study in the Discussion section as follows:*

*“Although the variable “smoking” between the two groups had a statistically significant difference, in the multivariable COX regression analysis, this variable was not found to be an independent risk factor for mortality of patients with acute respiratory distress syndrome related to COVID-19. However, smoking can increase CO_2_ in blood gas analysis; thus, this issue could be another study limitation.”*

*On the other hand, we agree with the reviewer that this study was conducted in patients with severe ARDS; therefore, this issue was indicated in the title as well as in the method section:*

*The title was changed to “The ∆Pv-aCO2/∆Ca-vO2 ratio as a predictor of mortality in patients with severe acute respiratory distress syndrome related to COVID-19”.*

*“Inclusion criteria were: (1) age >18 years, (2) any gender, (3) confirmed SARS-CoV-2 infection by reverse transcriptase polymerase chain reaction (RT-PCR), and (4) severe ARDS (PaO2/FiO2 ≤100 mmHg) defined according to Berlin criteria [18] with invasive mechanical ventilation (IVM).” (Section of Material and methods, Study design and patients).*

**5.** Were all patients treated with the same vasopressors? If it is not, could you add this to the method section? Because vasopressor types or using vasopressors can affect the MV time and mortality in critically ill patients.

***Reply:*** *All patients were treated with norepinephrine as the only vasopressor. This sentence was included in the Material and Methods Section in this new submission:*

*“All patients were intubated in the ICU and treated with norepinephrine as only vasopressor”.*

**6.** In the Conclusion section, there was a sentence starting with ‘This study is the first study regarding patients with ARDS secondary to SARS-CoV-2’. It would be better if you start the sentence with it has not shown yet instead of ‘this is the first study’.

***Reply:*** *The sentence starting with “This study is the first study regarding patients with ARDS secondary to SARS-CoV-2…” was changed to “The use of the ∆Pv-aCO2/∆Ca-vO2 ratio as a predictor of mortality in patients with severe ARDS secondary to SARS-CoV-2 has not yet been proven.” (Conclusion Section).*

6. PLOS authors have the option to publish the peer review history of their article ([what does this mean?](https://journals.plos.org/plosone/s/editorial-and-peer-review-process#loc-peer-review-history)). If published, this will include your full peer review and any attached files.

If you choose “no”, your identity will remain anonymous but your review may still be made public.

**Do you want your identity to be public for this peer review?** For information about this choice, including consent withdrawal, please see our [Privacy Policy](https://www.plos.org/privacy-policy).

Reviewer #1: No

Reviewer #2: No

Please let me know about above questions.

By signing this letter, we acknowledge that all the authors participated sufficiently to take public responsibility for its content. All of the authors have given their consent for submission to the journal. Further, we have no commercial associations which impact this work.

Best regards,

**Professor José Manuel Reyes**

Mexican Social Security Institute (IMSS)

E-mail: [jose.reyesr@imss.gob.mx](mailto:jose.reyesr@imss.gob.mx)
