# Supplementary material for: The ∆Pv-aCO2/∆Ca-vO2 ratio as a predictor of mortality in patients with severe acute respiratory distress syndrome related to COVID-19
Source: PLoS One. 2023 Sep 6;18(9):e0290272. doi: 10.1371/journal.pone.0290272 (PMC10482256; doi:10.1371/journal.pone.0290272)
Supplement: Attachment — Submitted filename: RESPONSE TO ACADEMIC EDITOR COMMENTS.docx [file pone.0290272.s002.docx]

March 07^th^, 2023

**Emily Chenette**

**Editor-in-Chief**

**Bora Çekmen**

**Academic Editor**

**PlosONE**

Dear Editor,

Enclosed, please find the revised version of the manuscript entitled: “**∆Pv-aCO2/∆Ca-vO2 ratio as predictor of mortality in patients with acute respiratory distress syndrome related to COVID-19**” with ID: **PONE-D-22-29034**, which we are submitting for consideration for publication in PLOS ONE.

We attended to all the suggestions of the ACADEMIC EDITOR and performed additional modifications to complete this work.

**RESPONSE TO ACADEMIC EDITOR' COMMENTS**

**ACADEMIC EDITOR:**

**1. There is a difference between the abstract and the main article with the changes you made in the conclusion part. This needs to be fixed again.**

***Reply:*** *Thank you very much for your comments. In this new submission, to eliminate the differences between the abstract and the main article with the changes previously made in the conclusion part, the conclusion was modified in the Abstract and Conclusion sections, as follows:*

*In the Abstract:*

*The sentence: Conclusions: “Patients with severe ARDS secondary to SARS-CoV-2 have increased ∆Pv-aCO2/∆Ca-vO2 ratio due to severe hypoxemia. Hence, ∆Pv-aCO2/∆Ca-vO2 ratio could be used as a predictor of COVID-19 mortality”* ***was changed to*** *“The ∆Pv-aCO2/∆Ca-vO2 ratio could be used as a predictor of mortality in patients with severe ARDS secondary to SARS-CoV-2.”*

*In the Conclusions*

*The sentence: “In patients with severe ARDS secondary to SARS-CoV-2, non-survivors have an increased ∆Pv-aCO2/∆Ca-vO2 ratio due to hypoxia secondary to severe hypoxemia, reflecting increased anaerobic metabolism. In contrast, circulatory blood flow alterations secondary to ischemic hypoxia are less relevant. The ∆Pv-aCO2/∆Ca-vO2 ratio >2.14 mmHg/mL is an independent risk factor for mortality (HR 1.17, 95% CI 1.06-1.29, p= 0.001) in this patient population. The ∆Pv-aCO2/∆Ca-vO2 ratio could help determine the prognosis”* ***was changed to*** *“The ∆Pv-aCO_2_/∆Ca-vO_2_ ratio >2.14 mmHg/mL is an independent risk factor for mortality (HR 1.17, 95% CI 1.06-1.29, p= 0.001) in patients with severe ARDS secondary to SARS-CoV-2. Hence, the ∆Pv-aCO_2_/∆Ca-vO_2_ ratio could help determine the prognosis of these patients.”*

**2. In the Abstract section, a few sentences of explanation are required about which parameters you are examining in the Method section. You must do it in a way that complies with the word count limit.**

***Reply:*** *In this new submission, the abstract was modified complying with the word count limit to include a few sentences explaining the parameters that were examined in the Material and Methods section (number of words: 273 words):*

*“Abstract*

*Objective: To evaluate the central venous-to-arterial carbon dioxide difference combined with arterial-to-venous oxygen content difference (∆Pv-aCO2/∆Ca-vO2 ratio) as a predictor of mortality in patients with COVID-19-related severe acute respiratory distress syndrome (ARDS).*

*Methods: Patients admitted to the intensive care unit with severe ARDS secondary to SARS-CoV-2, and invasive mechanical ventilation were included in this single-center and retrospective cohort study performed between April 18, 2020, and January 18, 2022. The tissue perfusion indexes (lactate, central venous oxygen saturation [ScvO2], and venous-to-arterial carbon dioxide pressure difference [∆Pv-aCO2]), anaerobic metabolism index (∆Pv-aCO2/∆Ca-vO2 ratio), and severity index (Simplified Acute Physiology Score II [SAPSII]) were evaluated to determine its association with the mortality through Cox regression analysis, Kaplan-Meier curve and receiver operating characteristic (ROC) curve.*

*Results: One hundred fifteen patients were included in the study and classified into two groups, the survivor group (n= 54) and the non-survivor group (n= 61). The lactate, ScvO2, ∆Pv-aCO2, and ∆Pv-aCO2/∆Ca-vO2 ratio medians were 1.6 mEq/L, 75%, 5 mmHg, and 1.56 mmHg/mL, respectively. The ∆Pv-aCO2/∆Ca-vO2 ratio (Hazard Ratio (HR)= 1.17, 95% confidence interval (CI)= 1.06-1.29, p= 0.001) was identified as a mortality biomarker for patients with COVID-19-related severe ARDS. The area under the curve for ∆Pv-aCO2/∆Ca-vO2 ratio was 0.691 (95% CI 0.598-0.774, p= 0.0001). The best cut-off point for ∆Pv-aCO2/∆Ca-vO2 ratio was >2.14 mmHg/mL, with a sensitivity of 49.18%, specificity of 85.19%, a positive likelihood of 3.32, and a negative likelihood of 0.6. The Kaplan-Meier curve showed that survival rates were significantly worse in patients with values greater than this cut-off point.*

*Conclusions: The ∆Pv-aCO2/∆Ca-vO2 ratio could be used as a predictor of mortality in patients with severe ARDS secondary to SARS-CoV-2.”*

**3. You should change the changes you made in the results section of the main text in the abstract section as well.**

***Reply:*** *The changes that were made in the results section of the main text are mentioned in the Abstract Section of this resubmission of the manuscript.*

**4. In the Abstract section, “Results” should be written instead of “Data synthesis”.**

***Reply:*** *In the Abstract section, “Data synthesis” was changed to “Results”.*

Thank you very much for your comments. Please let me know about above questions.

By signing this letter, we acknowledge that all the authors participated sufficiently to take public responsibility for its content. All of the authors have given their consent for submission to the journal. Further, we have no commercial associations which impact this work.

Best regards,

**Professor José Manuel Reyes**

Mexican Social Security Institute (IMSS)

E-mail: [jose.reyesr@imss.gob.mx](mailto:jose.reyesr@imss.gob.mx)
