# Supplementary material for: The ∆Pv-aCO2/∆Ca-vO2 ratio as a predictor of mortality in patients with severe acute respiratory distress syndrome related to COVID-19
Source: PLoS One. 2023 Sep 6;18(9):e0290272. doi: 10.1371/journal.pone.0290272 (PMC10482256; doi:10.1371/journal.pone.0290272)
Supplement: Attachment — Submitted filename: Response to reviewers Round 3.docx [file pone.0290272.s003.docx]

July 13^th^, 2023

**Emily Chenette**

**Editor-in-Chief**

**Bora Çekmen**

**Academic Editor**

**PlosONE**

Dear Editor,

Enclosed, please find the revised version of the manuscript entitled: “**The ∆Pv-aCO2/∆Ca-vO2 ratio as a predictor of mortality in patients with severe acute respiratory distress syndrome related to COVID-19**” **[PONE-D-22-29034R2] - [EMID:325f746b4f6fc97d]**, which we are submitting for consideration for publication in PLOS ONE.

We attended to all the suggestions of the Reviewer #3 and performed additional modifications to complete this work.

**RESPONSE TO REVIEWER'S COMMENTS**

**Reviewer #3: I thank the editor for sending me this manuscript for review and revision.**

**Interesting work with some points to be clarified, with the methods poorly expressed and explicated making the work difficult to reproduce and unclear in its development. The authors should be clearer on several points that, thoroughly explored would make the manuscript clear and comprehensive, without gray spots that could be misinterpreted.**

***Reply:*** *Thank you very much for your review and comments. We have made the necessary changes and modifications to the manuscript.*

**In any case, an interesting work to be reworked further but its results are not negligible.**

**Materials and Methods.**

**I would have some questions for the authors with respect to the inclusion and exclusion criteria and also for some subsequent points.**

**1. The authors state that patients with diseases that could affect hemoglobin level were excluded. But how come only those? As we see later in the formulas, diseases that can affect CO2 should also have been excluded, since they also lead to situations where CO2 is compensated by an adaptation over time in which the patient lives normally but with CO2 that could be "corrected."**

***Reply:*** *We agree with the reviewer. Although not previously mentioned, we also excluded patients with* *chronic obstructive pulmonary disease (COPD), known neuromuscular disease or known hyperbaric respiratory failure which could affect CO_2_ levels. We include these exclusion criteria in this new submission as follows (Page 4, Material and Methods Section):*

*(1) Patients with diseases that could affect the hemoglobin or CO2 levels as hematologic diseases, chronic obstructive pulmonary disease (COPD), known neuromuscular disease or known hyperbaric respiratory failure; (2) patients with an incomplete variable registry; or (3) pregnant were excluded from this study.*

2. A further claim made, later by the authors is that all patients were intubated in the ICU and treated with norepinephrine as the only vasopressor.

This statement assumes that all patients treated by the authors and examined did not need any other inotropic support. So this was an inclusion or exclusion criterion, depending on how we see the reasoning. Correct? All patients had adequate contractile function and never needed any inotropic support other than vasoconstrictor norepinephrine?

***Reply:*** *The sentence “All patients were intubated in the ICU and an treated with norepinephrine as the only vasopressor” is a typing mistake. In our study,* *all patients were intubated in the ICU but some of them received norepinephrine (n= 26, 22.60%) as the only vasopressor (Table 1). These patients did not require ionotropic support other than the vasoconstrictor norepinephrine. Thus, inotropic support was not an inclusion or exclusion criterion.*

*In this new submission (Page 4, Material and Methods Section), the sentence: “All patients were intubated in the ICU and an treated with norepinephrine as the only vasopressor” was changed to “**All patients were intubated in the ICU and some of them received norepinephrine (n= 26, 22.60%) as the only vasopressor. These patients did not require ionotropic support other than the vasoconstrictor norepinephrine.”*

**3. The authors state that an on-call critical care specialist scheduled the IVM, does this mean everything and does it mean nothing, how was the IVM "scheduled," were ARDS ventilation criteria met, was advanced monitoring (esophageal pressure, transpulmonary) used for monitoring, or is this not a procedure that usual in the ICU in question? Was ventilation guided by driving pressure or solely by plateau pressure? These are all details that are helpful in understanding, later also, the patient's aerobic and anaerobic consumption and assessment.**

**Other points, which I did not find in the methods, are both the sedation of the patients, how were they managed? And also the ventilatory modalities? When were these variables assessed? In the hyperacute phase or retrospectively? These points are important**

***Reply:*** *We agree with the reviewer that these points are important in order to provide further information. First, lung-protective mechanical ventilation was applied in the volume assist-controlled mode using the Puritan Bennett 840 ventilator (Medtronic; Carlsbad, California, USA), with the following settings: tidal volume of 6 mL/Kg predicted body wight, plateau pressure ≤27 cmH_2_O, and driving pressure ≤15 cmH_2_O.*

*Second, the patients were sedated using propofol and mechanical ventilation was started. After 30 min of ventilation in a supine positioning the ventilatory variables, including perfusion indexes and anaerobic metabolism, were assessed.*

*In this new submission, we included these points in the Material and methods Section (Pages 4-5) as follows:*

*“All the patients were sedated using propofol and mechanical ventilation was started. Lung-protective mechanical ventilation was applied in the volume assist-controlled mode using the Puritan Bennett 840 ventilator (Medtronic; Carlsbad, California, USA), with the following settings: tidal volume of 6 mL/Kg predicted body wight, plateau pressure ≤27 cmH_2_O, and driving pressure ≤15 cmH_2_O. After 30 min of ventilation in a supine positioning the ventilatory variables, including perfusion indexes and anaerobic metabolism, were assessed.”*

**4. Regarding the first point, it is important because depending on the level of sedation that is applied to the patient, the patient has more or less consumption than he needs. If we sedate or even curarize a patient we know that we conspicuously lower the patient's consumption and requirement, on the other hand a patient who is breathing independently, perhaps in "conflict" with the ventilator, or with a fever or ongoing sepsis needs more requirement because he or she has a higher "consumption."**

***Reply:*** *The propofol infusion was administered to maintain a Richmond Agitation-Sedation Scale (RASS) score of -3 (moderate sedation; patient had any movement in response to voice, but no eye contact) and overcome ventilator asynchrony, obtain a level of awake sedation, optimizing the patient´s respiratory status without effects on respiratory pattern, respiratory drive, and arterial and central venous blood gases.*

*This response was included in the Material and methods Section (Page 5, paragraph 2) of this new submission.*

**5. This also affects the ventilatory modalities adopted during the study and for the evaluation period, perhaps in the very early phase there is not this big consumption since the patient is put completely at "rest," conversely, during awakening and weaning we need more requirements. All these points should be considered by the authors always in this methods section, or at least adequately described.**

***Reply:*** *We agree with the reviewer. The level of sedation that is applied to the patient could affect the ventilatory variables adopted during the evaluation study. However, this evaluation was performed in the early phase (after 30 min of ventilation in a supine positioning) where there is not a big consumption since the patient is put completely at “rest”. This point was answered as follows:*

*“All the patients were sedated using propofol and mechanical ventilation was started. Lung-protective mechanical ventilation was applied in the volume assist-controlled mode using the Puritan Bennett 840 ventilator (Medtronic; Carlsbad, California, USA), with the following settings: tidal volume of 6 mL/Kg predicted body wight, plateau pressure ≤27 cmH_2_O, and driving pressure ≤15 cmH_2_O. After 30 min of ventilation in a supine positioning the ventilatory variables, including perfusion indexes and anaerobic metabolism, were assessed. The arterial and central venous blood gases were determined in the GEM® PREMIER™4000 with iQM® equipment.*

*The propofol infusion was administered to maintain a Richmond Agitation-Sedation Scale (RASS) score of -3 (moderate sedation; patient had any movement in response to voice, but no eye contact) and overcome ventilator asynchrony, obtain a level of awake sedation, optimizing the patient´s respiratory status without effects on respiratory pattern, respiratory drive, and arterial and central venous blood gases.”*

**Discussion**

**6. An interesting part that the authors could also analyze is the presence of significance between live and dead with respect to creatinine and bases. Which is well seen in the comparison between the two groups, then in the Cox Regression the creatinine does not become significant but the bases remain significant. Did the authors think that the renal part might also be involved in these mechanisms?**

***Reply:*** *Although creatinine and bases levels showed significant differences between the non-survivors and survivors (p< 0.05), only the base was statistically significant in the univariate Cox regression analysis. However, according to the multivariate Cox regression analysis the base had no significant association with the non-survivors. For this reason, we do not consider that the renal part might also be involved in these mechanisms. This response was included in the new submission (Discussion Section, Page 9, paragraph 3).*

**7. One thing that has not been taken into consideration by the authors is also the fact that patients are affected by hypoxia, and thus come to the hospital, after a period when the hemoglobin dissociation curve (p50) has shifted, allowing patients to better "tolerate" hypoxia as shown by many present studies. This aspect could also influence the outcome of the work. What do the authors think about this aspect? Did they take it into consideration?**

***Reply:*** *We agree with the reviewer. An important point is that the deterioration of lung function during SARS-CoV-2 infection induces alternative compensation mechanisms for oxygen uptake, such as the enhanced hemoglobin oxygen through a left shift of the oxygen dissociation curve, increasing perfusion, which modulates central venous blood gases [1]. Although the oxygen dissociation curve was not performed in this study, clinical relevance cannot be excluded. It needs further evaluation to determine their impact on the ∆Pv-aCO_2_/∆Ca-vO_2_ ratio in the prognostic of COVID-19 patients.*

*This response was included in this new submission in the Discussion Section (Page 11, paragraph 2).*

**Conclusion**

**8. I would be slightly softer, not stating that the ratio is an independent risk factor, but stating that in this study the ratio was found to be an independent risk factor, precisely also in light of the limitations of the study.**

***Reply:*** *We made the correction in the conclusion as suggested by the reviewer:*

*In this study the* *∆Pv-aCO_2_/∆Ca-vO_2_ ratio >2.14 mmHg/mL was an independent risk factor for mortality (HR 1.17, 95% CI 1.06-1.29, p= 0.001) in patients with severe ARDS secondary to SARS-CoV-2.*

Thank you very much for your comments. Please let me know about above questions.

By signing this letter, we acknowledge that all the authors participated sufficiently to take public responsibility for its content. All of the authors have given their consent for submission to the journal. Further, we have no commercial associations which impact this work.

Best regards,

**Professor José Manuel Reyes**

Mexican Social Security Institute (IMSS)

E-mail: [jose.reyesr@imss.gob.mx](mailto:jose.reyesr@imss.gob.mx)

**Reference**

1.	Böning D, Kuebler WM, Bloch W. The oxygen dissociation curve of blood in COVID-19. American Journal of Physiology-Lung Cellular and Molecular Physiology. 2021;321: L349–L357. doi:10.1152/ajplung.00079.2021
